# Retraction: MXene–PVA composite for arsenic removal from industrial wastewater: a combined DFT and experimental study

**DOI:** 10.1039/d6ra90151d

**Published:** 2026-10-23

**Authors:** Vu Thi Hoa

**Affiliations:** a Chemical Engineering Faculty, Industrial University of Ho Chi Minh City 700000 Vietnam Vu.hoa88@gmail.com

## Abstract

Retraction of ‘MXene–PVA composite for arsenic removal from industrial wastewater: a combined DFT and experimental study’ by Vu Thi Hoa, *RSC Adv.*, 2026, **16**, 2981–2995, https://doi.org/10.1039/d5ra09190j.

I, the author, hereby wholly retract this article after identifying concerns regarding the completeness, traceability, and variability of some of the data supporting the reported results, and therefore can no longer guarantee the reliability of the findings.

I, the author, am happy with the decision to retract this article.

Signed: Vu Thi Hoa, 7th October 2026

Retraction endorsed by Laura Fisher, Executive Editor, *RSC Advances*

